# Editorial: Advanced Technologies and Perspectives on Sustainable Microalgae Production

**DOI:** 10.3389/fbioe.2022.841261

**Published:** 2022-02-17

**Authors:** Muthu Arumugam

**Affiliations:** ^1^ Microbial Processes and Technology Division, CSIR-National Institute for Interdisciplinary Science and Technology, Thiruvananthapuram, India; ^2^ Academy of Scientific and Innovative Research (AcSIR), Gaziabad, India

**Keywords:** algae, sustainable food production, safe environment, carbon sequestration, omega-3 fatty acid food security

Algae are photoautotrophs like plants, produce their food by fixing atmospheric carbon dioxide in the presence of sunlight. They produce half of the total atmospheric oxygen, hence supporting all lifeforms on the earth. They can be unicellular or multicellular, microalgae or macroalgae and occur naturally in moist areas, freshwater, and marine environments. Being the primary producers in marine ecosystems, they form the base of most aquatic food webs. They multiply rapidly, have short generation time, and hence can spread across large areas in less time. They can withstand harsh environments without compromising survival due to their highly adaptive nature and can accumulate a wide range of products like fatty acids (oils), carbohydrates, chromophores (carotenoids, chlorophyll, phycobiliproteins), antioxidants, vitamins, enzymes, polymers, peptides, toxins, and sterols. These high-value products find diverse applications in biofuel production, food/feed supplements, nutraceuticals, cosmetics, natural colouring agents, and therapeutic and pharmaceutical fields. Apart from these, algae became extensively used in bioremediation, wastewater treatment, biochar and biofertilizers.

Microalgae has gained high interest in the global bioenergy sector as a third-generation biofuel feedstock with, potential to meet global transportation fuel demand. The algal biomass serves as sources of biofuels such as biodiesel, bioethanol, biohydrogen, biomethane, and gasoline. Algae-based fuels are carbon-neutral because they tend to not disturb carbon emission-fixation balance and could significantly reduce the rising threats of global warming, unlike coal-based fossil fuels. Microalgal strains with higher lipid and/or fermentable sugar content are exploited for biodiesel and bioethanol production. These strains are isolated, cultivated under controlled conditions of optimized pH, temperature, nutrients, and light in photobioreactors and open raceway ponds over large areas, followed by harvesting and downstream processing to obtain the required product. The natural oil content in algae comes in the range of 20–50 percent of its dry weight and the oil yield far exceeds that from terrestrial crops. However, algal cultivation requires higher maintenance and harvesting costs, and advanced technologies for conversion to biofuels. Achieving indefinite availability of algal biofuels in higher volumes for fulfilling future demands is a big challenge in terms of capital investment and technological support. This makes algal-based biofuel production not commercially viable and economically feasible when compared to other conventional biofuels.

Various cost-effective strategies for improving commercial viability of algae-based biofuels have been proposed, one of them being co-production of value-added products from algae. The algal biomass is a rich natural source of antioxidants, polyunsaturated fatty acids (PUFAs), vitamins, and minerals and has the potential to serve as nutrient-enriched supplements to food diets, in therapeutic, cosmetic, and pharmaceutical applications. Their supplementation in small quantities would be suffice for meeting adequate nutritional demands of the larger population. Microalgal PUFAs like omega-3-fatty acids [Docosahexaenoic acid (DHA) and Eicosapentaenoic acid (EPA)] and omega-6-fatty acids [Arachidonic acid and gamma-linolenic acid (GLA)] offer many health-promoting benefits and treatment of diseases related to the cardiac and vascular system, nervous system, arthritis, obesity, mental health, and various autoimmune disorders. The supplementation of these nutrient-rich algal sources into dietary intakes of poultry, aquaculture, animal feeds and human diets enhance food quality and overall health of the consuming population. This ensures greater food security to maintain the nutritional status of population, foreseeing a disease-free healthy future. Considering the reckless usage of fossil fuels and shrinking of cultivable land, the Alga, primary producer of aquatic ecosystem may gain a significant place in ensuring the food security, environment conservation by sequestering CO_2_ and liberation of oxygen in near future.

